# The electrophysiology of language production: what could be improved

**DOI:** 10.3389/fpsyg.2014.01560

**Published:** 2015-01-13

**Authors:** Vitória Piai, Stéphanie K. Riès, Robert T. Knight

**Affiliations:** Department of Psychology, Helen Wills Neuroscience Institute, University of California BerkeleyBerkeley, CA, USA

**Keywords:** EEG, ERP, speech, word production, response time

Recently, the field of spoken-word production has seen an increasing interest in the use of the electroencephalogram (EEG), mainly for event-related potentials (ERPs). These are exciting times to be a language production researcher. However, no matter how much we would like our results to speak to our theories, they can only do so if our methods are formally correct and valid, and reported in ways that allow replicability. Inappropriate practices in signal processing and statistical testing, when applied to our investigations, may render our conclusions invalid or non-generalizable. Here, we first present some issues in signal processing and statistical testing that we think deserve more attention when analysing data, reporting results, and making inferences. These issues are not new to electrophysiology, so our sole contribution is to reiterate them in order to provide pointers to literature where they have been discussed in more detail and solutions have been proposed. We then discuss other issues pertinent to our investigations of overt word-production because of the effects (and potential confounds) that speaking will have on the signal. Although we cannot provide answers to some of the issues raised, we invite researchers in the field to jointly work on solutions so that the topic of the electrophysiology of word production can thrive on solid grounds.

## Improving pre-processing

A common step in ERP analysis is filtering. In many studies, all we can find regarding the filtering procedure are cut-off values. However, this is incomplete information since a filter has other important parameters that affect the outcome of the filtering procedure. Different software will vary in their default values for these parameters. Researchers should not only try to understand how different filter parameters affect the signal studied (e.g., Widmann et al., 2014), but also at a minimum report the following in addition to the cut-off value (Picton et al., 2000; Gross et al., 2013): software used for filtering, filter type, order, and direction of the filter (forward, backward, or both), and whether any changes were made to default parameters.

Another common step is to define a pre-stimulus baseline period, which can then be used to normalize the rest of the signal. This pre-stimulus baseline provides a good indication of the signal-to-noise ratio (SNR) in the data. If an ERP difference post-stimulus is similar in magnitude as pre-stimulus differences, the post-stimulus difference is likely noise, not an effect induced by our manipulation (e.g., Woodman, 2010). Therefore, even if one does not apply baseline correction, the signal should always be displayed including a pre-stimulus interval so that the SNR can be evaluated.

## Improving statistical analysis

Results that cannot be explained by mere chance are highly informative for our theories. However, by using statistical tests inappropriately, we may make incorrect inferences regarding the probability of our results. An example is the well-known increased family-wise error rate (FWER, the probability of false positives amongst all multiple tests performed at some alpha-level) associated with the common practice of testing multiple time windows for significance (see Supplementary Material for an example). Alternatively, certain time points/windows may be selected for statistical testing on the basis of some criterion. However, a biased selection of this criterion also results in an inflation of false positives (Kilner, 2013). Under a different approach, successive univariate tests are conducted and effects are considered significant if the number of adjacent significant time points exceeds a pre-determined threshold (Guthrie and Buchwald, 1991). Piai et al. (2014) showed the problems associated with the incorrect determination of this threshold, leading to increased FWER. When possible, we should opt for statistical tests that provide nominal FWER control while maintaining statistical power, such as cluster-based statistics for example (Maris and Oostenveld, 2007; Pernet et al., 2014). Other valuable recommendations are provided in Allen et al. (2012) and Rousselet and Pernet (2011).

## Unsolved issues

For many years the dominant notion was that muscle activity associated with overt production would contaminate the EEG signal. Recently, that view has changed and the increasing number of ERP studies employing overt production is claimed to support the feasibility of combining EEG with overt speech. However, an increasing number in ERP studies employing overt production does not confirm that measuring ERPs with overt production is unproblematic. Moreover, even though it has been argued that “artifact-free brain responses can be measured up to at least 400 ms post-stimulus presentation” (Ganushchak et al., 2011, p. 5), the question we should ask is what constitutes an artifact in the context of overt production. Myogenic speech-related artifacts may precede speech onset by up to 500 ms, compromising the potentials recorded on the scalp (e.g., Brooker and Donald, 1980). If RTs differ consistently between conditions, the speech-related artifacts could also contaminate the pre-speech signal with consistent timing differences, resulting in an artifactual ERP effect.

Artifacts aside, another problem is physiological in nature. We are interested in which (and when) differences emerge in the waveforms time-locked to a stimulus as a function of our experimental manipulation. We know that our manipulation elicits a difference between conditions—an effect—in vocal response times (RTs). However, breathing and articulation are functions controlled by the brain. So RT differences are likely to be accompanied by systematic differences between the conditions in the relative timing of speech-related artifacts and of brain activity related to the control of speech that are independent of linguistic effects. This problem is well-known and has been mentioned, for example, by Luck (2005) in his rules of ERP experimental design and interpretation: “Be cautious when the presence or timing of motor responses differs between conditions” (p. 97).

Researchers have measured movement-related cortical potentials preceding mouth opening (Deecke et al., 1986; Yoshida et al., 1999) and speech-related breathing cortical potentials preceding phonation (Tremoureux et al., 2014). Importantly, these cortical potentials may precede mouth opening and phonation by 600 ms or more (Yoshida et al., 1999; Galgano and Froud, 2008). If conditions differ consistently with respect to when participants prepare to move their mouth, which is likely given any systematic RT differences, ERP differences between conditions could emerge as a function of these potentials. In this case, the effect is truly neural, yet not directly reflecting the cognitive function of interest. Finally, suppression of the auditory system may occur pre-speech onset (Ford et al., 2007; Wang et al., 2014). This raises the possibility that speech output efference copy affects the ERPs measured for conditions differing in RT since the timing of auditory suppression would systematically differ between conditions.

**Figure 1:**
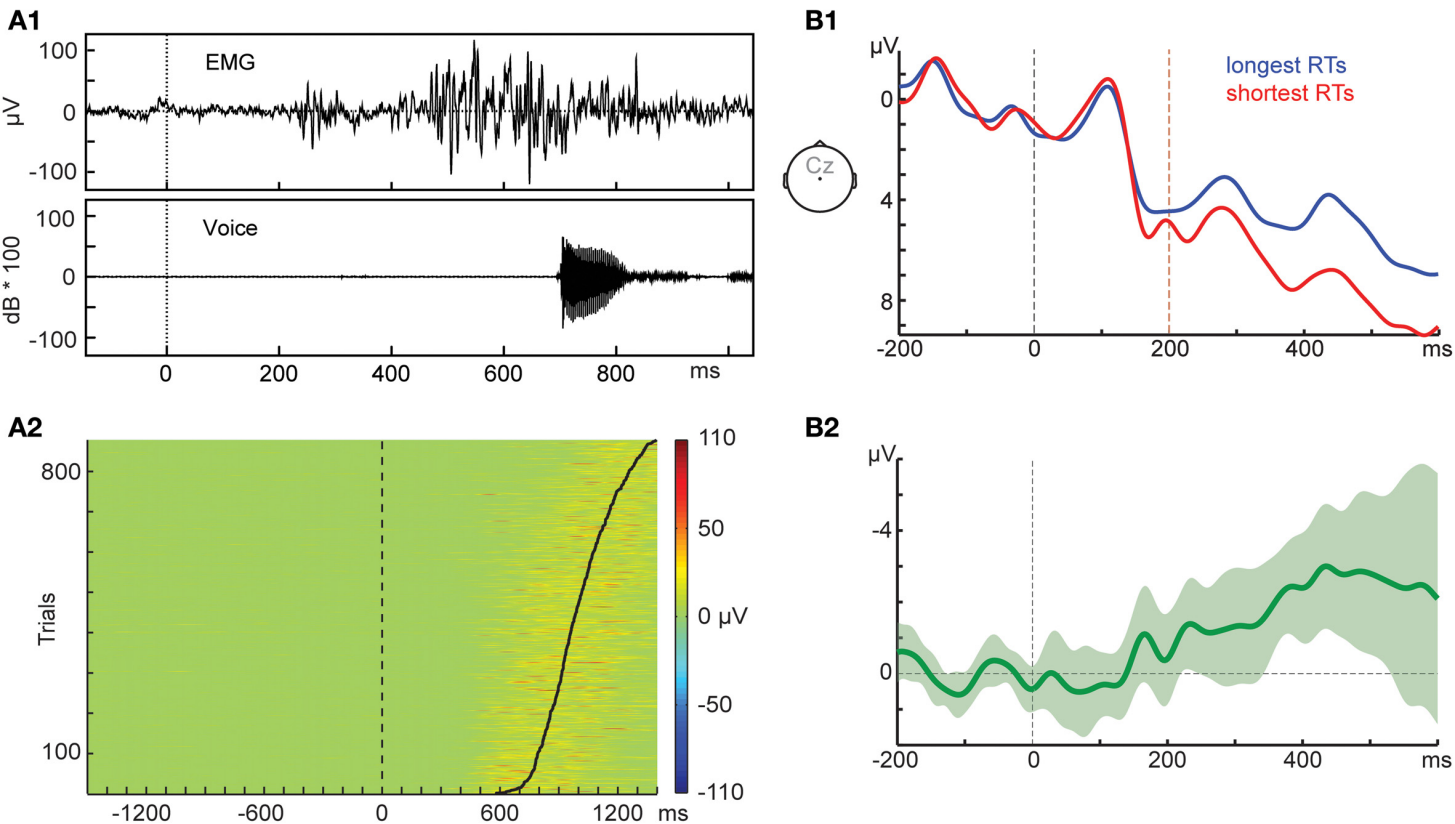
**(A1)** Amplitude of the electromyogenic (EMG) activity recorded from the risorius muscle and the corresponding acoustic signal for the pronunciation of the word *parc* in Experiment 1 of Riès et al. (2012). The task was single word naming with the visual word presented at the 0-ms time point. **(A2)** Single-trial EMG activity of 15 participants recorded from the orbicularis oris muscle sorted by picture naming time (solid black line). The task was picture naming with the picture presented at the 0-ms time point. **(B1)** Event-related potentials (ERPs) of 15 participants recorded during a picture-naming task. ERPs from the same condition were split by the participants' median picture naming time (RT). The picture was presented at the 0-ms time point (black dashed line). For reference, the orange dashed line indicates the 200-ms time point. The ERPs were filtered with a 20-Hz low-pass Butterworth filter of order 4 applied forward and backward using FieldTrip (Oostenveld et al., 2011). **(B2)** Difference wave. Shaded area indicates 95% confidence interval.

Electromyogenic (EMG) activity recorded from mouth muscles provides valuable information on this issue. EMG activity, either directly related to the articulation of the response or merely preparatory, can start as early as 250 ms after stimulus onset (Riès et al., 2012, 2014), as exemplified in Figure 1A1 for one word-naming trial. The lower panel (Figure 1A2) shows how EMG activity is consistently increased (indicated by the warmer colors) prior to speech onset (indicated by the solid black line) on the single-trial level (see Supplementary Material for details). Neural activity to move these muscles must precede the first measurable excitation on the muscle itself, so we cannot know whether potentials preceding speech are reflecting our cognitive manipulation only, or are already overlapping with the neural signals needed to control the mouth muscles.

Figure 1B provides another example of this issue. Participants' ERPs from one same picture-naming condition were split by their median RT, creating two surrogate conditions (mean longest RTs = 992 ms, mean shortest RTs = 729 ms). As Figure 1B1 shows, a simple difference in RT between two “conditions” can result in ERP differences of at least 1 μV starting as early as 200 ms. This early ERP difference is significant with various statistical tests (see Supplementary Material for details), *p*-values between 0.008 and 0.056. Note that dichotomizing a variable, as we do here, is not a recommended practice in statistics (MacCallum et al., 2002), so our median-split approach is only meant to illustrate this point, but should not be taken as a valid approach to investigate the relation between RTs and the electrophysiology of language production.

Of course one could argue that RTs are shorter or longer for some cognitive reason, so the differences shown in Figure 1B simply reflect cognitive processes associated with this slowing down. Moreover, the timing of this ERP “effect” could be taken as an indication that our manipulation tapped a certain cognitive process. The question is whether we should interpret the ERP effect in Figure 1B as reflecting a cognitive function of interest even though the ERPs come from the *same* cognitive manipulation. Rather, given that the observed ERP waveform is a sum of latent components, we should consider the possibility that our observed ERP effects are the net result of latent components reflecting a manipulated cognitive factor and latent components with consistent timing differences reflecting cortical activity related to low-level aspects of speaking, such as breathing and muscle control. In fact, this remark has been made very recently with respect to the breathing potentials preceding phonation: “the findings [… ] indicate the need to take the respiratory component of speech and its cortical determinants into account when conducting and interpreting such studies” (Tremoureux et al., 2014). The extent to which these issues may be especially pertinent to ERP components closer to articulation onset also deserves attention. Note that, although our example shows more positive-going ERPs for trials with shorter RTs, this relation should not be taken as a rule across studies. This is because different studies are likely to obtain different configurations of latent components reflecting breathing, muscle control, and cognitive factors, and these different configurations are likely to yield different observed ERP waveforms (e.g., Luck, 2005).

One may ask whether early effects are problem-free in this respect. However, the answer is complicated by the physiological issues described above in combination with technical issues. One of them may be caused by acausal filters (i.e., the filter applied forwards and then backwards). Due to this procedure, later slow components (possibly speech-related potentials and artifacts) can affect earlier parts of the signal (Acunzo et al., 2012), artificially creating an “ERP effect” that seems early enough to be the cognitive component of interest, rather than speech-related. The extent to which this factor could affect the signal that language-production researchers study is to our knowledge largely unknown. Additionally, if the low-pass filter is not appropriately designed for the data in question, speech-related cortical potentials and artifacts may end up smeared for tens of milliseconds before and after the event of interest (e.g., VanRullen, 2011; Widmann and Schröger, 2012), artificially creating differences that are early enough to seem related to the cognitive function of interest. Again, the extent to which this factor could affect the signals we study is largely unknown. In fact, the issues raised with respect to RT and breathing-pattern differences are not exclusive to spoken-word production. Timing differences in manual responding or in breathing and heart rate, and skin conductance (e.g., in studies on emotion) are likely to have some of the problems discussed here.

In conclusion, we need to consider that the ERP differences observed in conditions differing in RT are partly reflecting the relative difference in the timing of brain activity related to speaking (breathing and mouth movements) and other speech-related artifacts, in addition to our cognitive manipulation. We should also consider the possibility that our cognitive manipulation is *not necessarily* reflected in a stimulus-locked ERP component and that the ERP differences we observe reflect speech-related potentials only. Researchers could record mouth EMG activity and breathing rate in addition to scalp EEG to assess whether these pre-speech potentials are overlapping with the ERP effects observed over the scalp. These issues need to be addressed so that our field can move forward on a solid foundation.

### Conflict of interest statement

The Associate Editor, Dr. F-Xavier Alario, declares that despite having collaborated with author Dr. Stephanie K. Ries, the review process was handled objectively. The authors declare that the research was conducted in the absence of any commercial or financial relationships that could be construed as a potential conflict of interest.
