# Supplementary material for: The electrophysiology of language production: what could be improved
Source: Front Psychol. 2015 Jan 13;5:1560. doi: 10.3389/fpsyg.2014.01560 (PMC4292226; doi:10.3389/fpsyg.2014.01560)
Supplement: Supplementary file 1 [file Presentation1.PDF]

Supplementary Material

The electrophysiology of language production: what could be improved

Vitória Piai*, Stéphanie K. Riès, Robert T. Knight

Department of Psychology and Helen Wills Neuroscience Institute, University of California Berkeley, Berkeley, CA, USA

*** Correspondence:** Corresponding Author, Knight Lab, Helen Wills Neuroscience Institute, Department of Psychology, University of California Berkeley, 132 Barker Hall, Berkeley, California, 94720, USA.
vpiai@berkeley.edu

1. Supplementary Data

Simulation protocol for the statistical analysis with multiple time windows

Real EEG data were employed in the simulations (Piai, Roelofs and Maris 2014). In that study, participants read sentences and named pictures. For our purposes, the EEG segments comprising the first word of each sentence in the nonconstraining condition were used, ranging from 150 ms pre-stimulus to 600 ms post-stimulus (see Piai, Roelofs and Maris 2014 for details). Data from 14 subjects were used for whom on average 98 trials were available. The data were sampled at 500 Hz during recording (online band-pass filtered between 0.016–100 Hz). Furthermore, trials were low-pass filtered at cut-offs of 30 Hz and 15 Hz with a zero-phase Hamming windowed finite impulse response filter of order 100 (Matlab 2010b, fir1 function, default parameters, no zero-padding, one-pass, 53 dB stopband attenuation). Data from 6 channels (Cz, C3, C4, Pz, P3, P4) were averaged for each participant as to represent data from one channel.

The trials of the 14 participants were randomly partitioned into two sets and then averaged to form two participant-average ERPs, representing two surrogate conditions. The random partitioning of the data from the same condition ensures a true null hypothesis. Time-window averages were computed from the participant-average ERPs in time windows of 50 (from -100 to 600 ms, 14 windows), 100 ms (-100 to 600 ms, 7 windows), and 150 ms (from -150 to 600 ms, 5 windows). Paired-sample *t*-tests were conducted on each averaged time-window comparing the two surrogate conditions. This procedure was repeated 1,000 times. By calculating the proportion of random partitions that yielded a significant difference between the two surrogate conditions, we can assess the family-wise error rate of this approach. Under an alpha-level of .05, about 5% of the random partitions should yield a significant difference between the two conditions. The figure below shows that more than 5% of the partitions show a significant effect, attesting to the poor FWER control with increasing number of windows tested.

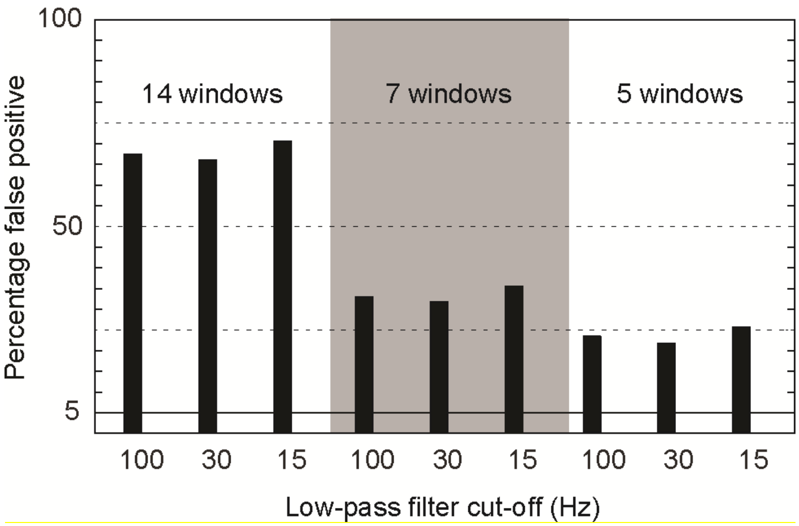

Figure. Percentage of false positives in the statistical simulations of averaged time-windows with 14 windows (left panel), 7 windows (middle panel), and 5 windows (right panel). The horizontal solid line indicates the 5% critical alpha-level. Additional horizontal dashed lines indicate the 25%, 50%, and 75% points. 100 Hz = data filtered only during recording (0.016–100 Hz online band-pass filter) with no additional offline low-pass filtering.

Electromyogenic activity: one trial (Figure 1A1, top panel)

One word-naming trial from the study of Riès et al. (2012) was selected for the figure (see for details Riès et al. 2012). The EMG signal shown in the figure was recorded from the risorius muscle with a sampling rate of 2048 Hz (DC to 268 Hz online filter). The speech signal was recorded with a piezoelectric microphone with a sampling rate of 22050 Hz. See the original article for further details on data preprocessing.

Electromyogenic activity: single-trial sorting by response time (Figure 1A2, lower panel)

EMG activity of the orbicularis oris muscle was recorded with a pair of surface Ag/AgCl-electrodes placed on the left upper and right lower corner of the mouth. During recording, the data were low-pass filtered by an anti-aliasing filter (300 Hz cutoff), digitised at 1200 Hz, and stored for offline analysis. Single-trial EMG activity was sorted by picture naming time (solid black line), using the erpimage function of EEGlab (Delorme and Makeig, 2004). The 0-ms time point indicates the timing of picture presentation.

Event-related potentials (Figure 1B)

From the nonconstraining condition of Piai, Roelofs and Maris (2014), the EEG was segmented between -200 ms pre-picture to 600 ms post-picture onset (the picture was presented at 0 ms). The signal was then filtered offline using FieldTrip (Oostenveld et al. 2011) with a 20 Hz low-pass Butterworth filter of order 4, applied forward and backward. For each participant, the median picture naming time (RT) was calculated. The single-trial EEG was then split into two conditions by this median RT and averaged, forming two conditions: longest and shortest RT. The participant's ERPs

were then baseline corrected by subtracting the mean signal between -200 to 0 ms from the signal post-picture onset.

For the statistical analysis, four tests were performed. In the first test, the signal from channel Cz was averaged within three time windows (post-picture onset): 200-250 ms, 200-300 ms, and 200-350 ms. Paired-sample *t*-tests were used for each time window, with the following results: 200-250 ms post-picture onset, $t(14) = 2.13$, $p = .05$; 200-300 ms, $t(14) = 2.08$, $p = .056$; 200-350 ms, $t(14) = 2.15$, $p = .05$. In a second test, the earliest time point showing the maximum difference between the two surrogate conditions was determined (228 ms) for channel Cz. Using a paired-sample *t*-test, the voltage at this time point was compared between the two surrogate conditions, $t(14) = 2.34$, $p = .035$ (for problems with this approach, see Kliner 2013). Thirdly, paired-sample *t*-tests between the two surrogate conditions were performed for each time sample and effects were considered significant if more than 11 successive significant t-tests were encountered (Guthrie and Buchwald, 1991; but see Piai, Dahlslätt and Maris, 2014). With this approach, two clusters were identified with more than 11 significant successive time points, one between 220-246 ms (14 successive significant time points) and another between 344-530 ms (94 successive significant time points). Finally, using cluster-based permutation (Maris and Oostenveld 2011) as implemented in FieldTrip (Oostenveld et al. 2011; default parameters, 3 neighbours per electrode on average), a significant cluster was detected between 344 and 540 ms, $p = .008$.

2. References

Delorme, A., and Makeig, S. (2004). EEGLAB: an open source toolbox for analysis of single-trial EEG dynamics. *J. Neurosci. Meth.* 134, 9-21.

Guthrie, D., and Buchwald, J. S. (1991). Significance testing of difference potentials. *Psychophysiology*. 28, 240-244.

Kilner, J. M. (2013). Bias in a common EEG and MEG statistical analysis and how to avoid it. *Clin. Neurophysiol.* 124, 2062–2063.

Maris, E., and Oostenveld, R. (2007). Nonparametric statistical testing of EEG- and MEG-data. *J. Neurosci. Meth.* 164, 177–190.

Oostenveld, R., Fries, P., Maris, E., and Schoffelen, J.-M. (2011). FieldTrip: Open source software for advanced analysis of MEG, EEG, and invasive electrophysiological data. *Comput. Intell. Neurosci.* 2011, 1–9.

Piai, V., Dahlslätt, K., and Maris, E. (2014). Statistically comparing EEG/MEG waveforms on the basis of successive univariate test statistics: How bad can it be? *Psychophysiology*.

Piai, V., Roelofs, A., and Maris, E. (2014). Oscillatory brain responses in spoken word production reflect lexical frequency and sentential constraint. *Neuropsychologia*. 53, 146-156.

Riès, S., Legou, T., Burle, B., Alario, A. and Malfait, N. (2012). Why does picture naming take longer than word reading? The contribution of articulatory processes. *Psychon. B. Rev.* 19, 955-961.
